# Differentiation-Dependent Regulation of Human Endogenous Retrovirus K Sequences and Neighboring Genes in Germ Cell Tumor Cells

**DOI:** 10.3389/fmicb.2018.01253

**Published:** 2018-06-15

**Authors:** Thomas Mueller, Claudia Hantsch, Ines Volkmer, Martin S. Staege

**Affiliations:** ^1^Department of Internal Medicine IV, Haematology/Oncology, Martin Luther University Halle-Wittenberg, Halle, Germany; ^2^Department of Surgical and Conservative Paediatrics and Adolescent Medicine, Martin Luther University Halle-Wittenberg, Halle, Germany

**Keywords:** endogenous retroviruses, germ cell tumor, differentiation, HERVK, PRODH, OCT4, LIN28A

## Abstract

Under physiological conditions, most human endogenous retroviruses (HERVs) are transcriptionally silent. However, re-activation of HERVs is observed under pathological conditions like inflammation or cancer. In addition to expression of HERV sequences, an impact of HERV-loci on expression of adjacent genes has been suggested as probably important patho-physiological mechanism. A candidate for such a gene is PRODH (proline dehydrogenase 1), which is located on chromosome 22 adjacent to HERVK-24. Germ cell tumors (GCTs) are known to express high level of HERVK sequences. In addition, non-seminomatous GCT are useful models to study HERV expression in the context of differentiation since they reflect aspects of cellular development during embryogenesis and usually contain different cell types. This is due to the embryonal carcinoma (EC) cells, which are the stem cell component of GCT. They are pluripotent, show high expression of pluripotency markers like OCT4 and LIN28A and can differentiate into either somatic derivatives (teratoma cells) or choriocarcinoma or yolk-sac tumor cells reflecting extra-embryonal differentiation. OCT4 is lost upon differentiation. We used GCT derived cell lines of varying differentiation stages to analyze expression of HERVK and PRODH. Differentiation status and cellular relationship of GCT cells was determined using microarray analysis and western blotting of the embryonic pluripotency markers OCT4 and LIN28A. The highest expression of HERVK was found in undifferentiated EC cells, which retain a stem cell phenotype and express both OCT4 and LIN28. In contrast, the lowest expression of HERVK was observed in somatic differentiated GCT cells which also lack OCT4 and LIN28A whereas GCT cells with differentiation characteristics of yolk-sac tumor expressed LIN28A but not OCT4 and showed intermediate level of HERVK. A similar pattern was found for PRODH. Differentiation of EC cells by siRNA mediated knock-down of OCT4 or treatment with differentiation inducing medium decreased expression of HERVK and PRODH. Treatment of differentiated GCT cells with 5′-azacytidine and trichostatin A increased expression of HERVK and PRODH, indicating that epigenetic mechanisms are responsible for altered expression of these genes. Our data suggest that HERVK expression is dependent on cellular differentiation stages regulated by epigenetic mechanisms, which can also affect expression of neighboring genes.

## Introduction

Human endogenous retroviruses (HERVs) are retroviral sequences that are permanently integrated into the human DNA and that are inherited from parents to the offspring like other genes. In addition to HERVs that are present in every individual, some HERVs are polymorphic and the presence or absence of these HERVs varies between individuals (Turner et al., 2001). Interestingly, these HERVs are particularly able to produce virus like particles (Boller et al., 2008). Re-activation of HERVs has been found in cancer patients. The patho-physiological function of this phenomenon is unclear but oncogenic transformation of cells by HERV gene products has been described (Boese et al., 2000; Galli et al., 2005; Argaw-Denboba et al., 2017; Lemaître et al., 2017). In addition, re-activation of HERV-like promoters has been shown to be involved in the aberrant expression of transformation associated genes in lymphoma cells (Lamprecht et al., 2010). Therefore, an impact of HERV-loci on expression of adjacent genes can be suggested as one probably important patho-physiological mechanism.

In this study we focused on a specific HERVK locus, which is referred to as ERVK-24 according to the nomenclature from Mayer et al. (2011). Formerly, this locus was described as HERV-K101 (Barbulescu et al., 1999) and c22_A (Ruprecht et al., 2008). ERVK-24 is located on chromosome 22 between the loci for proline dehydrogenase 1 (*PRODH*) and DiGeorge critical region 5 (*DGCR5*). *DGCR5* has been identified as chromosomal breakpoint in patients with DiGeorge syndrome (Sutherland et al., 1996). As *DGCR5* did not contain a functional open reading frame, it was suggested that expression of *DGCR5* might reflect a particular chromatin configuration that is required for regulation of adjacent genes (Sutherland et al., 1996). One candidate for such a gene is *PRODH*. *PRODH* is an evolutionarily conserved gene and a homolog of the *Drosophila* gene *sluggish A* (Gogos et al., 1999). Like PRODH, sluggish A is a mitochondrial protein and is involved in glutamate synthesis (Hayward et al., 1993). Mutations in *PRODH* are a cause of hyperprolinemia and a risk factor for schizophrenia (Bender et al., 2005).

ERVK-24 belongs to a group of HERVs with high expression in patients with germ cell tumors (GCTs) that are positive for antibodies against HERV-proteins (Flockerzi et al., 2008). It seems to be one of the transcriptionally most active HERV in GCT cells (Ruprecht et al., 2008). In addition to their high expression of HERVK sequences, GCTs, in particular non-seminomatous GCTs are useful models to study HERV expression in the context of differentiation processes since they can reflect some aspects of cellular development during embryogenesis. This is due to the pluripotent nature of embryonal carcinoma (EC) cells, which are the stem cell component of GCT. EC cells can be considered as the malignant counterpart of pluripotent embryonic stem cells, and show high expression of pluripotency markers like OCT4 (Looijenga et al., 2003; Sperger et al., 2003). They can differentiate into either somatic derivatives leading to teratoma tissue or into tissues like choriocarcinoma and yolk sac tumor reflecting an extra-embryonic differentiation (Oosterhuis and Looijenga, 2005). OCT4 is lost during differentiation. Therefore, GCT are usually composed of undifferentiated EC cells and variously differentiated cell types (Oosterhuis and Looijenga, 2005).

In the present paper we analyzed expression of HERVK and PRODH in cell lines of GCT with varying differentiation stages and upon induction of differentiation in undifferentiated cells. In addition, differentiated cells were treated with agents modifying DNA methylation and histone acetylation to investigate epigenetic mechanisms, which are known to be involved in both differentiation processes and inactivation of HERVs.

## Materials and Methods

### Cell Lines and Cell Culture

The following human GCT cell lines were used: H12.1 and H12.5 (Casper et al., 1987), H12.1D (Mueller et al., 2006), 1411HP (Vogelzang et al., 1985), GCT72 and GCT27 (Pera et al., 1987), 1777NRpmet, 2102EP, 833K, and NTera2-D1 (Bronson et al., 1980, 1983; Andrews et al., 1996). The cell lines 1777NRpmet, 1411HP, and 833K were kindly provided by Prof. Peter W. Andrews (University of Sheffield, United Kingdom). The H12.1 and H12.5 were established in the former group of Prof. H.-J. Schmoll (University Hospital Halle, Germany) and belong to our lab. The cell lines GCT72 and GCT27 were kindly provided by Prof. Martin F. Pera (Monash University, Australia, at the time of shipping). The NTera2-D1 was kindly provided by Dr. Heiko van der Kuip (University of Tübingen, Germany).

The Hodgkin lymphoma (HL) cell lines L-1236, L-428, L-540, KM-H2, and HDLM-2 (Schaadt et al., 1979; Diehl et al., 1982; Drexler et al., 1986; Kamesaki et al., 1986; Wolf et al., 1996) were purchased from the German Collection of Microorganisms and Cell Cultures, Brunswick, Germany.

All cell lines were cultured in RPMI-1640 (Invitrogen, Karlsruhe, Germany) supplemented with 10% fetal calf serum, 100 U/mL penicillin, and 100 μg/mL streptomycin at 37°C in a humidified atmosphere with 5% CO_2_. For induction of differentiation of H12.1 cells, cells were treated with 10 μM retinoic acid and harvested after 5 days. For re-induction of HERVK expression, 1777NRpmet cells were treated with 5′-azacytidine (4 μM) and trichostatin A (10 nM) and harvested after 5 days.

### Gene Expression Analysis

We used published cell lines and commercially available RNA from anonymous sources for gene expression analysis. RNA from cell lines was isolated using Trizol reagent (Invitrogen, Karlsruhe, Germany) following the manufacturer’s protocol. Probable DNA contamination was removed by treatment with DNase (Roche, Mannheim, Germany). In addition, RNA from human placenta from anonymous donors was obtained from Becton-Dickinson (Heidelberg, Germany). RNA (2 μg) were transcribed into cDNA using oligo-dT12-18 primers (Promega, Mannheim, Germany) and polymerase chain reaction (RT-PCR) was performed. The following primer combinations were used: actin beta (ACTB): 5′-GGC ATC GTG ATG GAC TCC G-3′, 5′-GCT GGA AGG TGG ACA GCG A-3′; HERVK primer combination a (HERVKa): 5′-CCT GCA GTC CAA AAT TGG TT-3′, 5′-GCA ATG CAA CTC CTG CTA CA-3′; HERVK primer combination b (HERVKb): 5′-TTC TGC TGG TGA GAG CAA GA-3′, 5′-TGG ACA CAG CAC ATG TTT CA-3′; glyceraldehyde 3-phosphate dehydrogenase (GAPDH): 5′-CCA TGG AGA AGG CTG GGG-3′, 5′-CAA AGT TGT CAT GGA TGA CC-3′; proline dehydrogenase 1 (PRODH): 5′-GAG GCT TTG AGA AGC CAG TG-3′, 5′-GGT ATT GCT TGT CCC GCT TA-3′. The PCR conditions were: 94°C, 30 s; 60°C, 30 s; 72°C, 45 s (35 cycles). The HERVK primers bind to the following genome coordinates: HERVKa:NC_000022.11:18945350-18945369 and NC_000022.11:18946285-18946304; HERVKb:NC_000022.11: 18946101-18946120 and NC_000022.11:18947034-18947049. The reverse primer from this combination has two mismatches with the current genome version. The primers should also be able to amplify additional HERVK elements. However, the very high expression of ERVK-24 in comparison to other elements seem to favor amplification of this locus as proved by sequencing of polymerase chain reaction products (see Supplementary Material). Absence of DNA contamination was tested randomly by using RNA without reverse transcription as template for PCR. See the Supplementary Material for an example. PCR products were subjected to agarose gel electrophoresis in the presence of ethidium bromide. Real-time quantitative RT-PCR (qRT-PCR) was performed using the Maxima^TM^ SYBR Green qPCR Master Mix (Fermentas, Sankt Leon-Rot, Germany) using the following conditions: 94°C, 45 s; 60°C, 45 s; 72°C, 60 s (40 cycles).

Global gene expression in GCT cells was analyzed using Affymetrix HG_U133A arrays (Affymetrix, Santa Clara, CA, United States). Arrays were processed essentially as described (Staege et al., 2004). In short, biotinylated cRNA was prepared by *in vitro* transcription after synthesis of double-stranded cDNA. After fragmentation of cRNA and hybridization, signals were detected with streptavidin-phycoerythrin and signals were enhanced by using goat-anti-streptavidin antibodies. Arrays were washed and stained with a GeneChip Fluidics Station 400 and scanned with a GeneArray Scanner G2500A. Affymetrix cell files were processed using Robust Multi-array Average (RMA) algorithm with Expression Console 1.1 (Affymetrix). GCT associated genes were identified on the basis of Wilks’ Lambda score (WLS) by using MAFilter (Winkler et al., 2012). WLS was used descriptively without significance calculation for filtering probe sets with high signal intensities in GCT cells in comparison to normal cells. For this end, WLS was calculated as quotient of the variance in the total group of samples and the variance in the group of normal tissues alone. Microarray cell files have been submitted to the Gene Expression Omnibus (GEO) data base (Accession No. GSE113423). For comparative analysis, published microarray data from a panel of normal tissues [normal body atlas (NBA)] from the GEO data base (GSE2361) were used (Ge et al., 2005). Cluster analysis and visualization was performed with Genesis (Sturn et al., 2002).

### Sequencing and Bioinformatical Analyses

Polymerase chain reaction products were purified with NucleoSpin Gel and PCR Clean-up (Machery-Nagel, Düren, Germany). Sequencing of PCR products was performed using the BigDye Terminator v1.1 Cycle Sequencing Kit (Life Technologies, Austin, TX, United States). The sequences were analyzed with BLAST (Altschul et al., 1990). Open reading frames in the intergenic region between PRODH and DGCR5 were identified by using getorf^1^. Long terminal repeats (LTRs) were identified using RepeatMasker^2^.

### Western Blot Analysis

Cells were harvested by trypsiniziation, rinsed twice with PBS and lysed in RIPA buffer (50 mM Tris-HCl pH 8.0, 100 mM NaCl, 0.5% NP40, 0.5% DOC, 0.5% SDS) supplemented with a protease inhibitor cocktail (Sigma, St. Louis, MO, United States). Insoluble components were removed by centrifugation and protein concentrations were measured (BIO-RAD protein assay, Bio-Rad, Hercules, United States). After boiling for 5 min in SDS-loading buffer (500 mM Tris-HCl pH 6.8; 10% glycerol, 2% SDS, 5% 2-mercaptoethanol, 0.05% bromophenol blue), 20 μg protein per lane was separated by SDS-PAGE and electroblotted onto nitrocellulose transfer membrane (Whatman, Maidstone, United Kingdom). Equal protein loading was controlled by Ponceau S staining (Sigma, St. Louis, MO, United States). Membranes were blocked with 5% non-fat dry milk in PBST for 1 h and probed for 2 h with the primary antibodies diluted in PBST/5% milk followed by incubation with secondary HRP-conjugated antibodies. Proteins were visualized by enhanced chemiluminescence (Carl Roth, Karlsruhe, Germany). The following primary antibodies were used: OCT4: sc-5279 mouse monoclonal C-10; β-actin: sc-1615 goat polyclonal C-11 (both from Santa Cruz Biotechnology, Santa Cruz, CA, United States); LIN28A: #3978 rabbit polyclonal (Cell Signalling). Horseradish peroxidase (HRP)-conjugated anti-goat, anti-mouse and anti-rabbit IgG (all from Santa Cruz Biotechnology, Santa Cruz, CA, United States) were used as secondary antibodies.

### siRNA-Mediated Protein Knock-down

For siRNA mediated protein knock-down of OCT4, cells were transfected with OCT4-specific siRNA or control-siRNA (both from Santa Cruz Biotechnology, United States). Transfection of siRNA was performed by the Nucleofector^®^-technology (Amaxa Biosystems, Germany). Cells (2 × 10^6^) were suspended in 100 μl transfection buffer (Amaxa Biosystems, Germany) and combined with 1 μg siRNA. After reaction in the Nucleofector^®^-system, the transfected cell suspension was diluted in growth media, seeded in 6 well plates and incubated for indicated times. OCT4 knock-down was confirmed by western blot analysis.

## Results and Discussion

**FIGURE 1:**
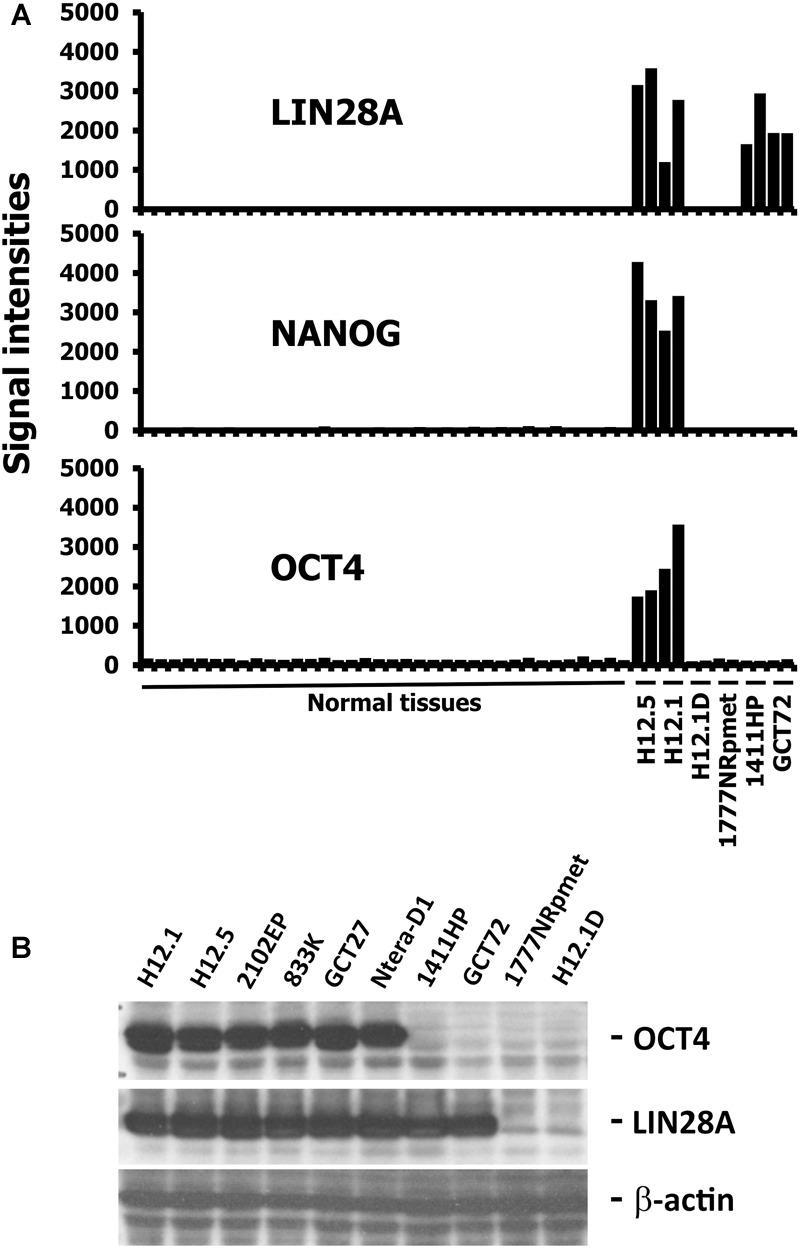
Expression of stem cell markers in GCT cells. **(A)** Gene expression in GCT cell lines was assessed by DNA microarray analysis. Two independent samples per cell line were analyzed. Gene expression in GCT was compared with gene expression in a panel of normal tissues (Ge et al., 2005). Genes with high expression in GCT were filtered by using MAFilter. Probe sets with a WLS > 10 were considered to be GCT specific. Presented are signal intensities (arbitrary units) for probe sets with specificity for the indicated stem cell markers. The following normal tissues are included (from left to right): heart, thymus, spleen, ovary, kidney, skeletal muscle, pancreas, prostate, small intestine, colon, placenta, bladder, breast, uterus, thyroid, skin, salivary gland, trachea, cerebellum, brain, fetal brain, adrenal gland, bone marrow, amygdala, caudate nucleus, corpus, hippocampus, thalamus, pituitary gland, spinal cord, testis, liver, stomach, lung, fetal lung, fetal liver. **(B)** Western blot analysis of pluripotent stem cell markers OCT4 and LIN28A. In addition to H12.1 and H12.5, four other cell lines representing the undifferentiated, pluripotent EC cell type were analyzed: 2102EP, 833K, GCT27, NTera-D1.

To investigate cellular relationship, we analyzed the gene expression pattern of six GCT cell lines with varying differentiation stages in comparison to a panel of normal tissues (NBA) from the GEO database (Ge et al., 2005). Microarray data were filtered for up-regulated genes on the basis of WLS by using MAFilter and 1,104 probes sets were identified with a WLS > 10 indicating up-regulation of the corresponding genes in GCT cells. Among the strongest up-regulated genes we found typical markers of pluripotent stem cells like *LIN28A, NANOG*, and *OCT4*. Based on the expression pattern of *LIN28A, NANOG* and *OCT4*, three groups of GCT cells could be defined (**Figure 1A**). Group 1 included the cell lines H12.1 and H12.5, which represent the undifferentiated, pluripotent EC cell type. These GCT cells are characterized by the expression of all three genes. Group 2 included the cell lines GCT72 and 1411HP, which have characteristics of differentiation toward yolk-sac tumor. These cells have lost expression of *NANOG* and *OCT4* but still express *LIN28A*. Group 3 included the cell line 1777NRpmet and the H12.1D, which is an *in vitro* differentiated, stable derivative of the EC cell line H12.1. These cells have lost expression of all three stem cell markers. The differential gene expression patterns were confirmed by western blot analysis of OCT4 and LIN28A (**Figure 1B**) indicating that both markers are useful to define the three groups of GCT cells.

**FIGURE 2:**
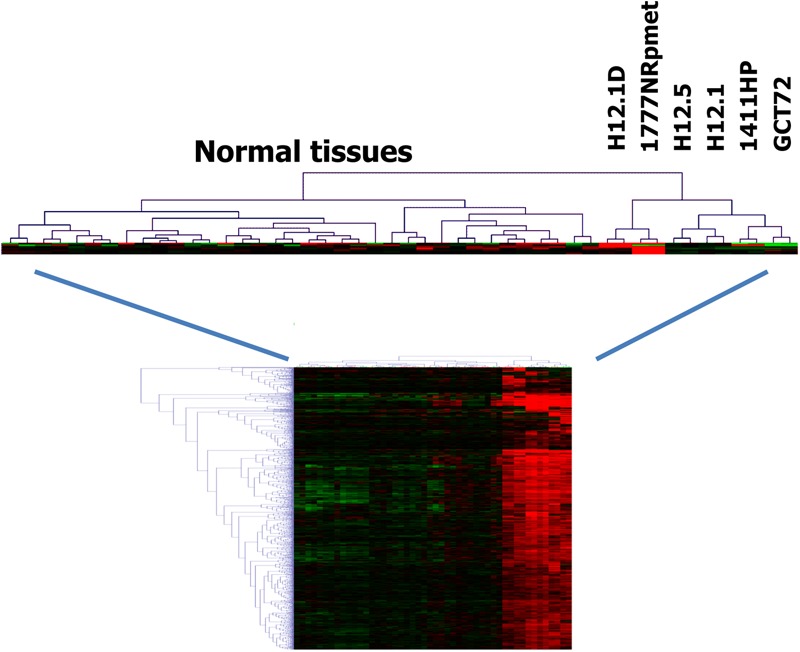
Relationship of GCT cells (i). Gene expression in GCT cell lines was assessed by DNA microarray analysis using two samples per cell line. Gene expression in GCT was compared with gene expression in a panel of normal tissues (Ge et al., 2005). Genes with high expression in GCT were filtered by using MAFilter. Probe sets with a WLS > 10 were considered to be GCT specific. Presented is a cluster analysis using these probe sets. Manhattan distance was used as distance metric. Signal intensities from microarray analysis were log2-transformed and median centered. Green indicates low expression, red high expression.

**FIGURE 3:**
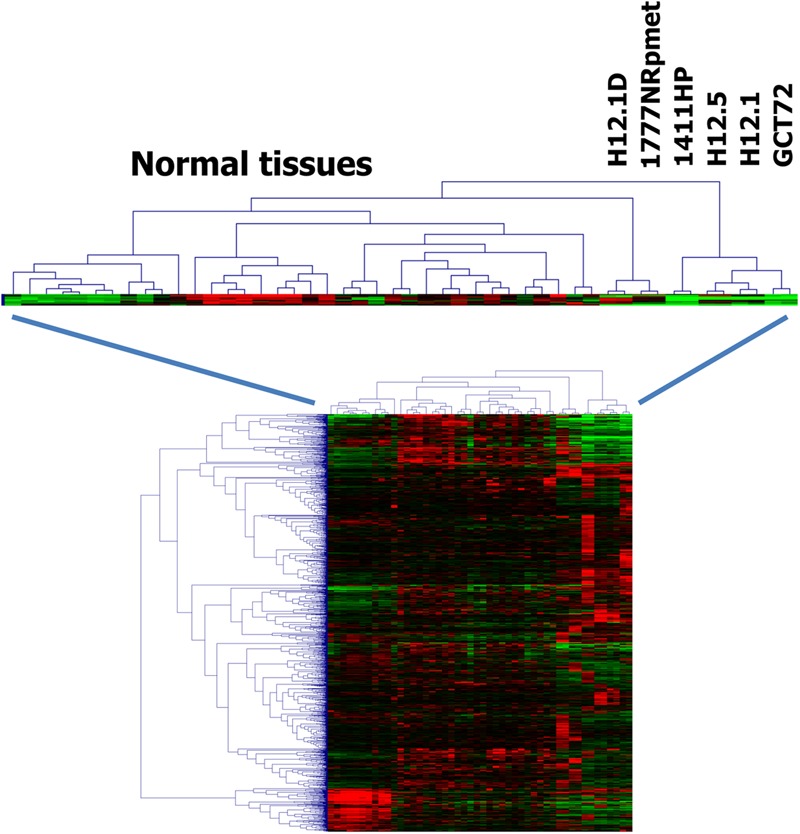
Relationship of GCT cells (ii). Gene expression in GCT cell lines was assessed by DNA microarray analysis using two samples per cell line. Gene expression in GCT was compared with gene expression in a panel of normal tissues (Ge et al., 2005). For each cell line individual specific genes were identified by dividing the mean signal intensity of these cell lines by the 85th percentile of the signal intensities in all other GCT cell lines. Only probe sets that showed a quotient of greater than two were analyzed further. Presented is a cluster analysis. Manhattan distance was used as distance metric. Signal intensities from microarray analysis were log2-transformed and median centered. Green indicates low expression, red high expression.

Cluster analysis indicated that the gene expression profile of cells from group 3 have greater similarity with normal somatic tissues than the gene expression profiles of the other groups (**Figure 2**) indicating a teratoma-like, somatic differentiation lineage of these cells. This similarity could also be seen in cluster analysis when we used probe sets that were filtered for cell line-specificity (**Figure 3**). For this end, we divided the mean signal intensity of each cell line (which in our case is identical to the 50th percentile) by the 85th percentile of the signal intensities in all other GCT cell lines. Using the 85th percentile has the advantage that outliers from these cell lines have only low impact on the calculated ratios. Cell line specificity was considered if this ratio was greater than 2. Based on this filtering criterion, a total of 1,315 probe sets showed cell line specificity. Cluster analysis using these probe sets as data points revealed again and more clearly the higher similarity between normal somatic tissues and 1777NRpmet and H12.1D cells. Interestingly, it also revealed that among the cells with yolk-sac tumor characteristics, GCT72 cells are closer related to pluripotent H12.1/H12.5 cells than 1411HP cells (**Figure 3**).

Together, gene expression analysis and western blotting could define three groups of GCT cells: (i) OCT4+/LIN28+ undifferentiated pluripotent, (ii) OCT4-/ LIN28+ differentiated toward yolk-sac tumor, and (iii) OCT4-/ LIN28- somatic differentiated.

**FIGURE 4:**
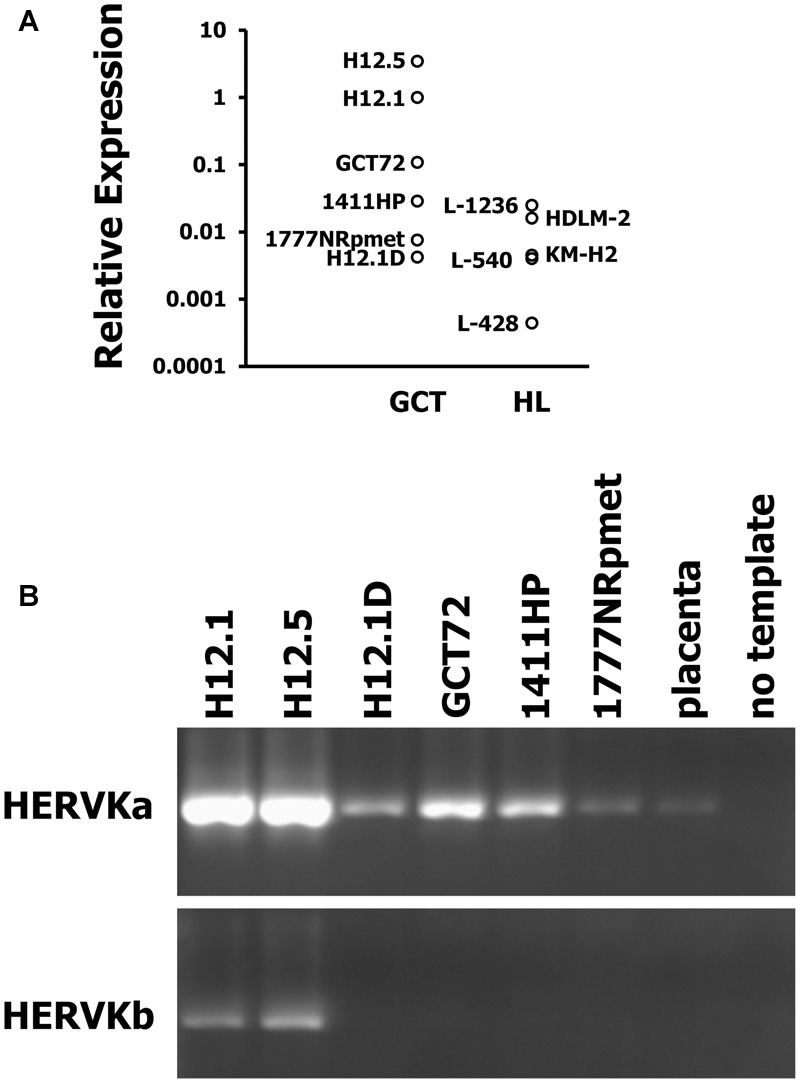
High expression of HERVK in GCT. Expression of HERVK in GCT cell lines was assessed by **(A)** quantitative RT-PCR (qRT-PCR) and **(B)** conventional RT-PCR with primers amplifying ERVK-24env. For qRT-PCR, HERVK specific primers were used and β-actin was used as housekeeping control. Expression of HERVK in H12.1 cells was set as 1 and relative expression was calculated according to the 2^-ΔΔCt^-method (Livak and Schmittgen, 2001). For conventional RT-PCR two different primer combinations were employed. cDNA from placenta was used as control.

**FIGURE 5:**
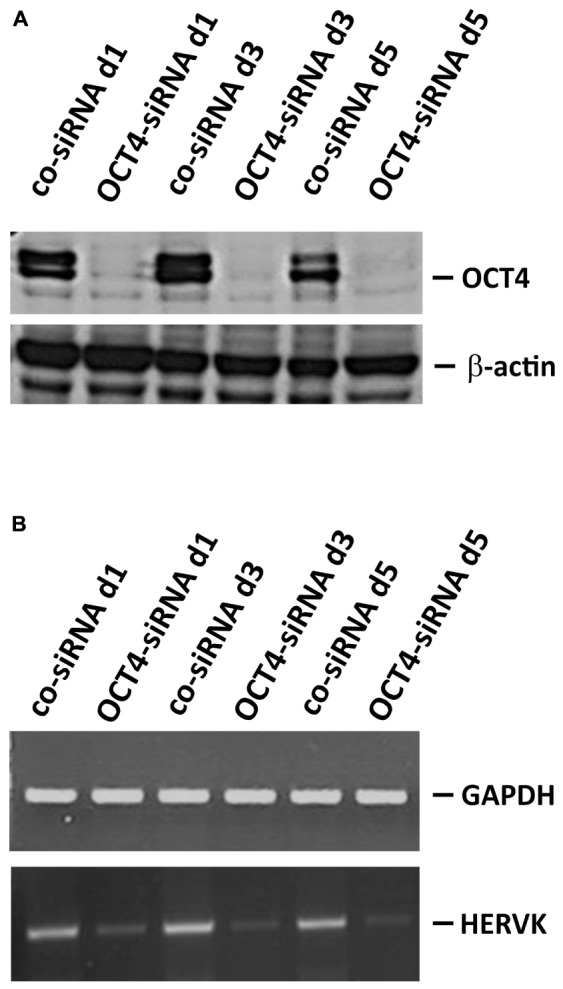
Repression of HERVK upon induction of differentiation in pluripotent GCT cells. Induction of differentiation in pluripotent H12.1 cells was performed by siRNA mediated knock-down of OCT4. Cells were analyzed after 24, 72, and 120 h by western blotting to prove knock-down of OCT4 **(A)** and by RT-PCR for HERVK expression **(B)**.

Germ cell tumors are known for their high expression of endogenous retroviruses. Therefore, we tested expression of HERVK in GCT cell lines by conventional and quantitative RT-PCR. For comparison we used HL cell lines. As shown in **Figure 4A**, GCT cell lines showed higher expression of HERVK than HL cell lines. Although reactivation of HERVs in HL cells has been described, the expression in HL cells is low in comparison to GCT at least for HERVK. Next we asked whether the different differentiation status of our GCT cell lines might have an impact on HERVK expression. Among GCT cells expression of HERVK was particularly high in undifferentiated pluripotent EC cell lines H12.1 and H12.5 (**Figure 4B**). Cells with characteristics of yolk-sac tumor (GCT72 and 1411HP) showed intermediate expression whereas somatically differentiated tumor cells (1777NRpmet, H12.1D) expressed lowest levels (**Figure 4B**). To prove a direct link between differentiation processes and HERVK expression, we performed siRNA mediated knock-down of OCT4 in pluripotent H12.1 cells which induces differentiation in those cells. As shown in **Figure 5**, induction of differentiation rapidly led to repression of HERVK.

**FIGURE 6:**
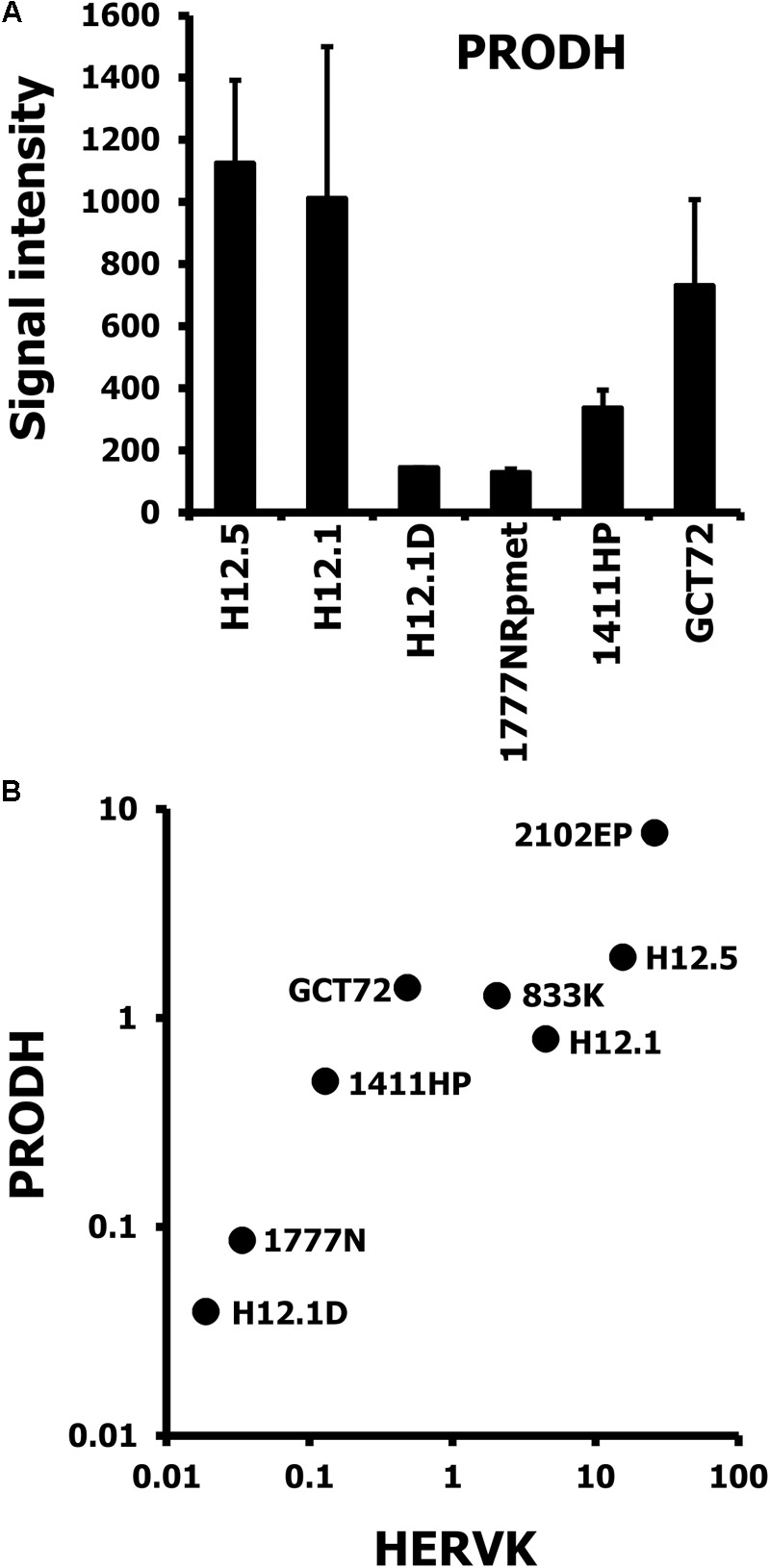
Correlative expression of HERVK and PRODH in GCT cells. **(A)** Gene expression in GCT cell lines was assessed by DNA microarray analysis. Two independent samples per cell line were analyzed. Presented are signal intensities (arbitrary units) for probe sets with specificity for PRODH as means ± SD. **(B)** Presented are results from quantitative RT-PCR analysis with primers specific for HERVK and PRODH. cDNA was prepared from GCT cell lines and β-actin was used as house-keeping control. Two additional cell lines (2102EP, 833K), which represent the undifferentiated, pluripotent EC cell type were included to support the correlation and to analyze differences among this group of GCT cells.

Sequencing of PCR products indicated that the primers used for PCR amplify preferentially ERVK-24 (see Supplementary Material). ERVK-24 is located between the loci for PRODH and DGCR5 (see Supplementary Material for the topography of the complete locus including the position of PCR amplicons). Organization of the locus suggests that PRODH and ERVK-24 might be regulated by a bi-directional promoter. We asked whether ERVK-24 and the neighboring PRODH might be co-regulated. Analysis of PRODH based on our microarray data showed a similar expression pattern of PRODH as observed for HERVK regarding the three groups of GCT cells (**Figure 6A**). Notably, GCT72 yolk-sac tumor cells had similar high PRODH expression as pluripotent H12.1 cells. Next we performed combined RT-PCR analysis of HERVK and PRODH in our GCT cell panel and found a correlation between PRODH and HERVK expression (**Figure 6B**). The characteristic higher PRODH expression in GCT72 among the cells with yolk-sac tumor differentiation could be reproduced. Therefore, PRODH and HERVK expression pattern of GCT72 confirmed the cluster analyses and suggest that it is more closely related to pluripotent H12.1/H12.5 cells than 1411HP.

**FIGURE 7:**
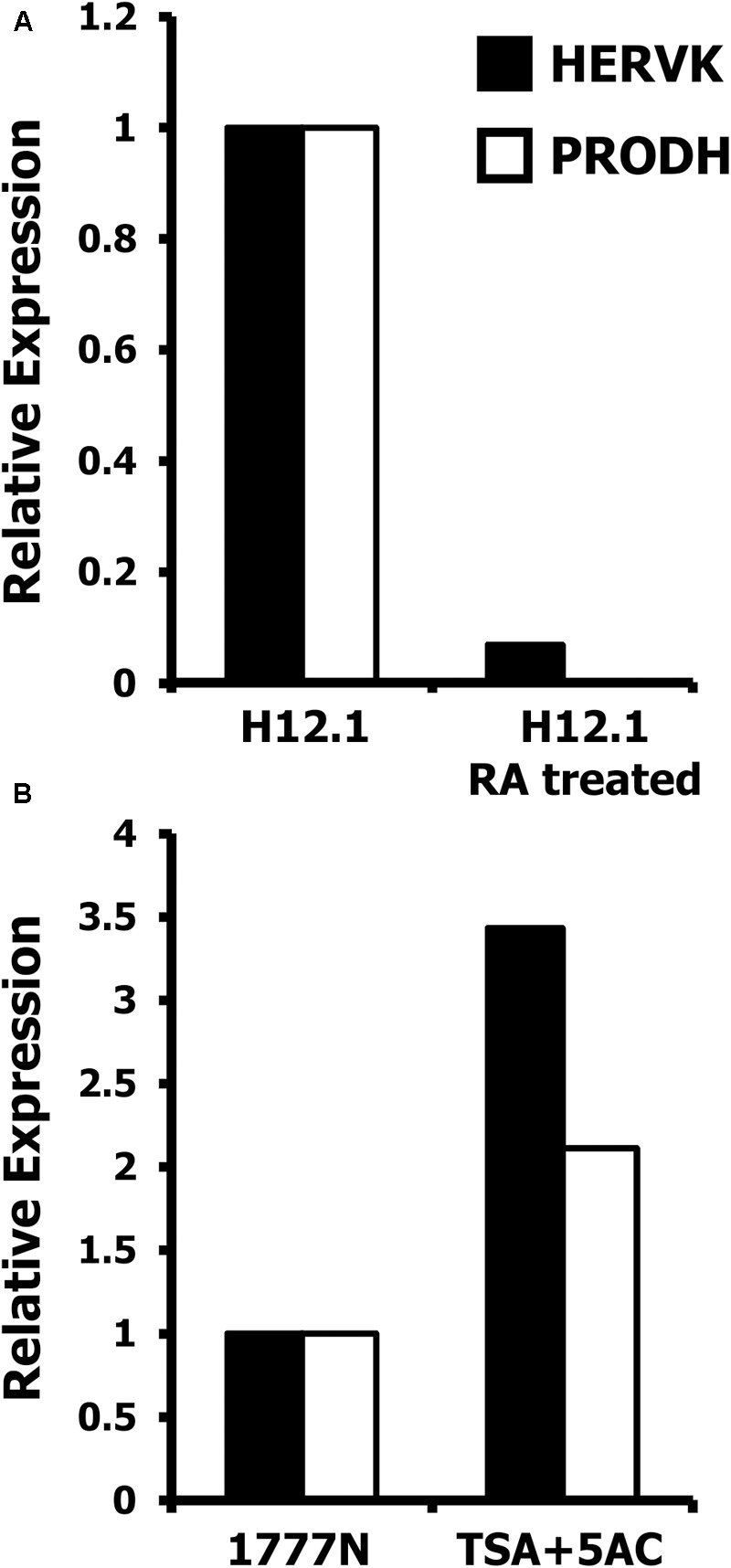
Epigenetic regulation of HERVK and PRODH in GCT cells. Presented are results from quantitative RT-PCR analysis with primers specific for HERVK and PRODH. cDNA was prepared from GCT cell lines and β-actin was used as house-keeping control. Expression of HERVK in un-treated cells was set as 1 and relative expression was calculated according to the 2^-ΔΔCt^-method (Livak and Schmittgen, 2001). **(A)** Pluripotent H12.1 cells were treated with retinoic acid to induce differentiation. **(B)** Somatically differentiated 1777NRpmet cells were treated with a combination of 5′-azacytidine and trichostatin A to induce re-expression of HERVK and PRODH.

To further investigate a possible differentiation dependent co-regulation of HERVK and PRODH, pluripotent H12.1 cells were treated with differentiation inducing retinoic acid. As shown in **Figure 7A**, induction of differentiation led to repression of HERVK and was accompanied by down-regulation of PRODH. Next we asked whether HERVK could be re-induced in cells with low expression, e.g., somatic differentiated cells. As shown in **Figure 7B**, treatment of 1777NRpmet cells with 5′-azacytidine and trichostatin A increased expression of HERVK. Interestingly, this was accompanied by induction of PRODH expression. Together these data demonstrate a differentiation-dependent and epigenetically-regulated expression of HERVK and suggest co-regulation of PRODH expression.

Expression of HERV sequences has been observed in different diseases including cancer. It remains unclear whether HERV expression is directly involved in pathogenesis or whether HERV expression is only an epi-phenomenon of altered gene regulation under pathological conditions. More recently, it was shown that activation of an endogenous retroviral LTR-like promoter is responsible for the expression of growth factor receptors in cancer cells (Lamprecht et al., 2010). However, the reasons for the aberrant activation of such promoters in cancer cells require further investigation. In the present paper we analyzed the expression of the PRODH/ERVK-24 locus. PRODH has been identified as a putative tumor suppressor gene (Liu et al., 2008, 2010). On the other hand, knock-down of PRODH decreases the viability of oxidized low-density lipoprotein (OxLDL)-treated cancer cells (Zabirnyk et al., 2010). OxLDL induce PRODH-dependent autophagy which may explain some of the effects of PRODH, because limited autophagy is a cell survival factor whereas excessive autophagy promotes cell death (Degenhardt et al., 2006).

In general, a large number of human genes are regulated by bi-directional promoters (Trinklein et al., 2004) and highly active HERV promoters might serve as bi-directional promoters (Domansky et al., 2000). The chromosomal organization of the PRODH/ERVK-24 locus together with our expression data in GCT suggests that both genes are co-regulated. Interestingly, expression of *PRODH* in germ line cells is evolutionarily highly conserved since germ line stem cells from *Drosophila* express high amounts of the *PRODH* homolog *sluggish A* (Kai et al., 2005). These data suggest that expression of *PRODH* is a feature of cells with an embryonic phenotype. The co-expression of ERVK-24 together with *PRODH* might be a consequence of the active chromatin state in GCT. Whether the expression of ERVK-24 and PRODH has consequences for the tumor cell biology requires further investigation.

## Conclusion

In addition to direct effects of HERV expression, co-regulation of neighboring genes should be considered as possible mechanism for HERV-associated diseases. This co-regulation can be associated with differentiation processes regulated by epigenetic mechanisms, as we have shown using GCT cell lines reflecting different stages of development.

## Author Contributions

TM and MS designed the study and wrote the paper. All authors performed experiments, analyzed the data, and approved the final version of the paper.

## Conflict of Interest Statement

The authors declare that the research was conducted in the absence of any commercial or financial relationships that could be construed as a potential conflict of interest.

## Footnotes

1 http://www.hpa-bioinfotools.org.uk/pise/getorf.html

2 http://www.repeatmasker.org/
