# Supplementary material for: Differentiation-Dependent Regulation of Human Endogenous Retrovirus K Sequences and Neighboring Genes in Germ Cell Tumor Cells
Source: Front Microbiol. 2018 Jun 15;9:1253. doi: 10.3389/fmicb.2018.01253 (PMC6013571; doi:10.3389/fmicb.2018.01253)

Differentiation-dependent regulation of human endogenous retrovirus K sequences and neighbouring genes in germ cell tumour cells

Thomas Mueller, Claudia Hantsch, Ines Volkmer, Martin S. Staege

Supplementary Material

1. Supplementary Figure 1. Prove of absence of relevant DNA contamination in RNA isolated from tumour cells. Page 2

2. Supplementary Figure 2. Schematic presentation of the PRODH-DGCR5 region on human chromosome 22. Page 3

3. Sequence and BLAST results of PCR product from H12.1 cells with primer combination HERVKa Page 4-5

4. Sequence and BLAST results of PCR product from H12.1 cells with primer combination HERVb Page 6-7

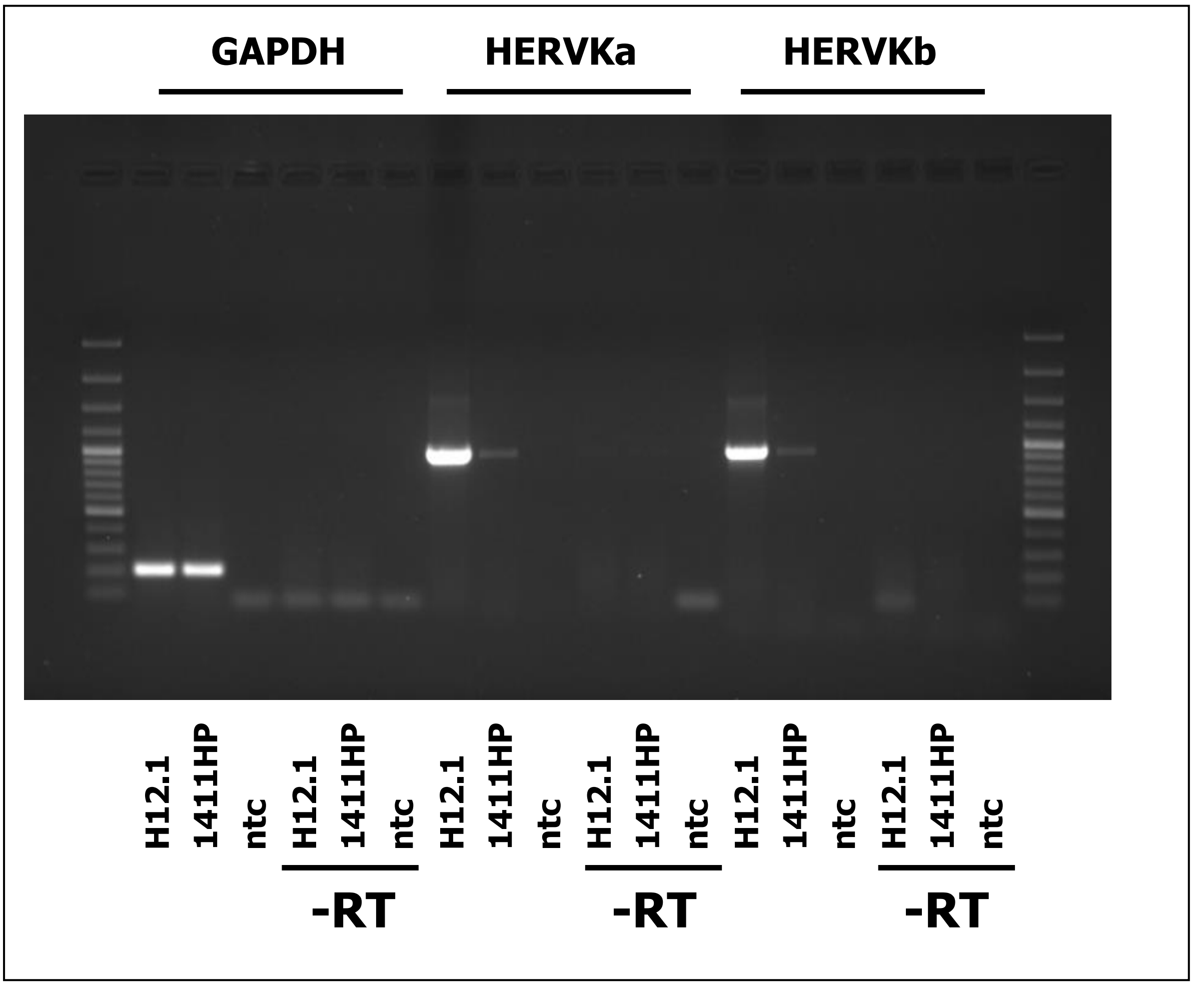

Supplementary Figure 1. Prove of absence of relevant DNA contamination in RNA isolated from tumour cells. RNA was isolated from indicated tumour cells, transcribed into cDNA and used as template for PCR with indicated primer combinations. In addition, RNA was processed without addition of reverse transcriptase (-RT) and used as target for PCR; ntc: no template control. GAPDH serves as housekeeping control and showed similar expression in both cell lines. HERVK signals were stronger in H12.1 cells than in 1411HP cells. Without reverse transcription, amplification was nearly undetectable.

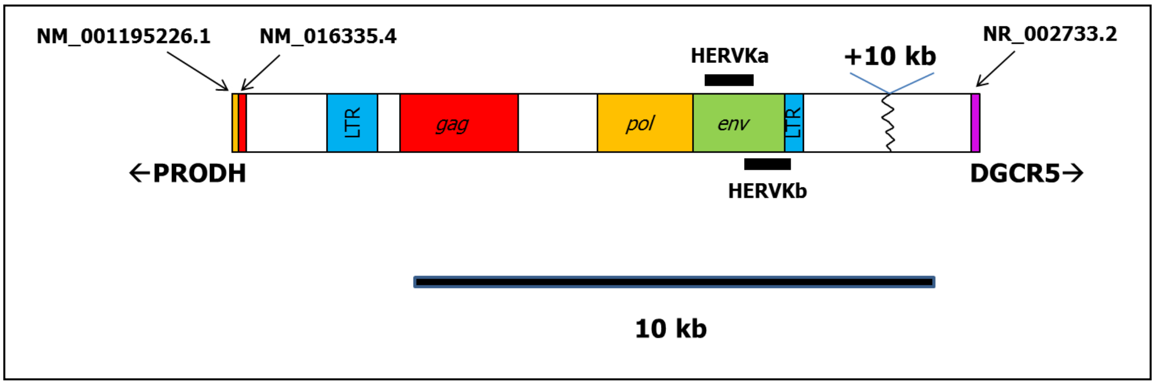

Supplementary Figure 2. Schematic presentation of the PRODH-DGCR5 region on human chromosome 22. For identification of ERV open reading frames, the intergenic region between PRODH and DGCR5 (accession number NC_000022.11:18936553-18970498) was scanned with getorf (http://www.hpa-bioinfotools.org.uk/pise/getorf.html) for large (>1 kb) open reading frames. The predicted protein sequences were analysed with BLAST (https://blast.ncbi.nlm.nih.gov/Blast.cgi) in order to assign the open reading frames to the typical viral gene products. LTR were identified with RepeatMasker (http://www.repeatmasker.org/). Only the positions of the two LTR sequences flanking the HERV are shown. According to RepeatMasker, Both LTRs are from the same family LTR5. The position of the PCR amplicons generated with primer combinations HERVKa and HERVKb as well as the start positions of transcripts from PRODH and DGCR5 are shown. For PRODH, two different start positions of the mRNA have been described.

Sequence of PCR product from H12.1 cells with primer combination HERVKa (without primer sequences):

```
GCACCCGTAGCTGTGACTGCAATTAATCCCATAATCACtGCAATTAAAGTAAAAATGAATCTTTTGGATCTATTTAAAACACC
TTTTAATACTTCAGTCAAAATATGGACGGATGGTGAGGCCTCCCACGGTCGGTCCATGGACACAAGGATCCACACGCCCTCTC
TTGCTCTCACCAGCAGAATACGGTGTTGCCAATTAAAAGTTGAATCAATGCAAGTAAGCAATCTACAGTTTTCACAGGTTATA
GTCTGGGAGTCTGGTTTAATAACTATATTTCCTACAACTAGCATATAAGGGGGCTTTACGCAACTTTGTAAAGGAAGTGTTAG
ACTGGAATTTAGGTCGACAGTATAAAATGGCTTACGATCTCTTGTTTCTAAAGTTTGATTTCCAGACCAAATTCTAATGTGGT
GTGAGGCCACAGTAAGCCTCCATAATTCTGGATGTTCAGGACCAGAAACAGGACTTATTATTTTTGGTCTTGGGGTAGAGATT
CCTTTTTCTCCCCATTCCCAAGGGTAGAAAGACTGCAATTTTTTATGCTTATGTTTGTCTAAACTTTCTGTTAAGTCGCTATC
AACAGCTGGACTCACTTGTGCACTTGGACATGACTGAGTTTGTCCTGAGCAATTGTGGTAGAATTGACCTCGAGGTGCCCAAT
CTATAATAGTTCCGAATTCATTGTTTTGTAATATCACCGCACTATTGGCCACACATTCTTCCCAAACTAAAACTTCTGTATTT
TTTGATTCTTTGGGAATTTCCTTGGGGCAAGGTTTCCCTTTAGGTCTAAATTTTAATGATCTTTGATAAGGAAAGTCTTGTAA
ATAATTTACCCGTGGCCTGAGTGACATCCCGCTTACCATGTGATAAGTGAATCTACTGATGGGACTGACAATAGGTACTTCTA
CC
```

Blast result; best hit: Homo sapiens chromosome 22, GRCh38.p12 Primary Assembly (NC_000022.11); Identities 100%, Gaps 0%:

```
Query  1         GCACCCGTAGCTGTGACTGCAATTAATCCCATAATCACTGCAATTAAAGTAAAAATGAAT  60
                 ||||||||||||||||||||||||||||||||||||||||||||||||||||||||||||
Sbjct  18946284  GCACCCGTAGCTGTGACTGCAATTAATCCCATAATCACTGCAATTAAAGTAAAAATGAAT  18946225
Query  61        CTTTTGGATCTATTTAAAACACCTTTTAATACTTCAGTCAAAATATGGACGGATGGTGAG  120
                 ||||||||||||||||||||||||||||||||||||||||||||||||||||||||||||
Sbjct  18946224  CTTTTGGATCTATTTAAAACACCTTTTAATACTTCAGTCAAAATATGGACGGATGGTGAG  18946165
Query  121       GCCTCCCACGGTCGGTCCATGGACACAAGGATCCACACGCCCTCTCTTGCTCTCACCAGC  180
                 ||||||||||||||||||||||||||||||||||||||||||||||||||||||||||||
Sbjct  18946164  GCCTCCCACGGTCGGTCCATGGACACAAGGATCCACACGCCCTCTCTTGCTCTCACCAGC  18946105
Query  181       AGAATACGGTGTTGCCAATTAAAAGTTGAATCAATGCAAGTAAGCAATCTACAGTTTTCA  240
                 ||||||||||||||||||||||||||||||||||||||||||||||||||||||||||||
Sbjct  18946104  AGAATACGGTGTTGCCAATTAAAAGTTGAATCAATGCAAGTAAGCAATCTACAGTTTTCA  18946045
Query  241       CAGGTTATAGTCTGGGAGTCTGGTTTAATAACTATATTTCCTACAACTAGCATATAAGGG  300
                 ||||||||||||||||||||||||||||||||||||||||||||||||||||||||||||
Sbjct  18946044  CAGGTTATAGTCTGGGAGTCTGGTTTAATAACTATATTTCCTACAACTAGCATATAAGGG  18945985
Query  301       GGCTTTACGCAACTTTGTAAAGGAAGTGTTAGACTGGAATTTAGGTCGACAGTATAAAAT  360
                 ||||||||||||||||||||||||||||||||||||||||||||||||||||||||||||
Sbjct  18945984  GGCTTTACGCAACTTTGTAAAGGAAGTGTTAGACTGGAATTTAGGTCGACAGTATAAAAT  18945925
Query  361       GGCTTACGATCTCTTGTTTCTAAAGTTTGATTTCCAGACCAAATTCTAATGTGGTGTGAG  420
                 ||||||||||||||||||||||||||||||||||||||||||||||||||||||||||||
Sbjct  18945924  GGCTTACGATCTCTTGTTTCTAAAGTTTGATTTCCAGACCAAATTCTAATGTGGTGTGAG  18945865
Query  421       GCCACAGTAAGCCTCCATAATTCTGGATGTTCAGGACCAGAAACAGGACTTATTATTTTT  480
                 ||||||||||||||||||||||||||||||||||||||||||||||||||||||||||||
Sbjct  18945864  GCCACAGTAAGCCTCCATAATTCTGGATGTTCAGGACCAGAAACAGGACTTATTATTTTT  18945805
Query  481       GGTCTTGGGGTAGAGATTCCTTTTTCTCCCCATTCCCAAGGGTAGAAAGACTGCAATTTT  540
                 ||||||||||||||||||||||||||||||||||||||||||||||||||||||||||||
Sbjct  18945804  GGTCTTGGGGTAGAGATTCCTTTTTCTCCCCATTCCCAAGGGTAGAAAGACTGCAATTTT  18945745
Query  541       TTATGCTTATGTTTGTCTAAACTTTCTGTTAAGTCGCTATCAACAGCTGGACTCACTTGT  600
                 ||||||||||||||||||||||||||||||||||||||||||||||||||||||||||||
Sbjct  18945744  TTATGCTTATGTTTGTCTAAACTTTCTGTTAAGTCGCTATCAACAGCTGGACTCACTTGT  18945685
Query  601       GCACTTGGACATGACTGAGTTTGTCCTGAGCAATTGTGGTAGAATTGACCTCGAGGTGCC  660
                 ||||||||||||||||||||||||||||||||||||||||||||||||||||||||||||
Sbjct  18945684  GCACTTGGACATGACTGAGTTTGTCCTGAGCAATTGTGGTAGAATTGACCTCGAGGTGCC  18945625
Query  661       CAATCTATAATAGTTCCGAATTCATTGTTTTGTAATATCACCGCACTATTGGCCACACAT  720
                 ||||||||||||||||||||||||||||||||||||||||||||||||||||||||||||
Sbjct  18945624  CAATCTATAATAGTTCCGAATTCATTGTTTTGTAATATCACCGCACTATTGGCCACACAT  18945565
Query  721       TCTTCCCAAACTAAAACTTCTGTATTTTTTGATTCTTTGGGAATTTCCTTGGGGCAAGGT  780
                 ||||||||||||||||||||||||||||||||||||||||||||||||||||||||||||
Sbjct  18945564  TCTTCCCAAACTAAAACTTCTGTATTTTTTGATTCTTTGGGAATTTCCTTGGGGCAAGGT  18945505
Query  781       TTCCCTTTAGGTCTAAATTTTAATGATCTTTGATAAGGAAAGTCTTGTAAATAATTTACC  840
                 ||||||||||||||||||||||||||||||||||||||||||||||||||||||||||||
Sbjct  18945504  TTCCCTTTAGGTCTAAATTTTAATGATCTTTGATAAGGAAAGTCTTGTAAATAATTTACC  18945445
Query  841       CGTGGCCTGAGTGACATCCCGCTTACCATGTGATAAGTGAATCTACTGATGGGACTGACA  900
                 ||||||||||||||||||||||||||||||||||||||||||||||||||||||||||||
Sbjct  18945444  CGTGGCCTGAGTGACATCCCGCTTACCATGTGATAAGTGAATCTACTGATGGGACTGACA  18945385
Query  901       ATAGGTACTTCTACC  915
                 |||||||||||||||
Sbjct  18945384  ATAGGTACTTCTACC  18945370
```

Blast result; top hits:

Chromosome	Start	Stop	identities
NC_000022.11	18946284	18945370	915/915
NC_000001.11	75381890	75380976	907/915
NC_000011.10	101702960	101702046	907/915
NC_000008.11	7499443	7499443	905/915
NC_000003.12	185564116	185565030	904/915
NC_000005.10	156659274	156660188	904/915

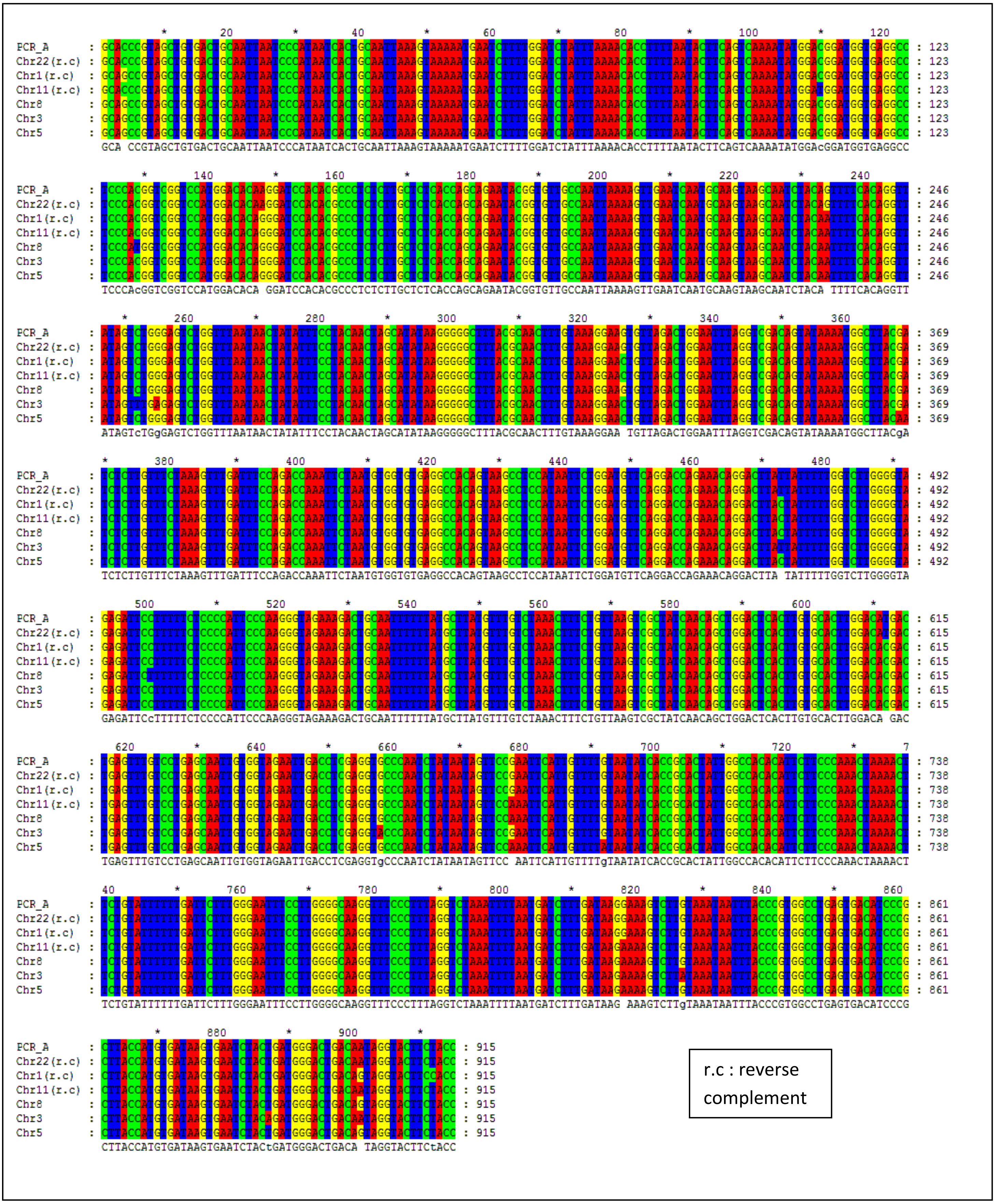

Sequence of PCR product from H12.1 cells with primer combination HERVKb (without primer sequences):

```
GAGAGCACGGGGTTGGGGGTAAGGTTATAGATTAACAGAaTCTCAAGGCAGAAGAATTTTTCTTAGCACATAACAAAATGGAG
TCTCCTATGTCTACTTCTTTCTACACAGACACAGTAACAATCTGATCTCTCTTGCTTTTCCCCACATTTCCCCCTTTTCTTTT
CGACAAAACCGCCATCGTCATCATGGCCCGTTCTCGATGGTCGCTGTCTCTTCGGAGCTGTTGGGTACACCTGCAGACTAACA
ACAGACAAAACAGGCACACAAGGATTAATATGAGATTTATAATCGTAGTACTTCCAATGGTCTTAACCCAAGTGACAGGGTTA
AGATTTGCGAGGCCATCAGCAACTCCTGCAATTGCCTCAGTTCCTGGCACCAAATTTAAATGGGCTTTTGATGCTTCGAAAAT
TTGTTCTTTTAATTTGGAAATGTCTAAAGTGAGATTATCTTCTCTTCCCTGTAGATGGCGTCTAACCATGTCCCAGTGATGCT
CAGACTCATTATAAATTTGGGGTGTAATACAAAAATCTGACGTATTCCAGTCACATTGTAACTGGAAACGATGTTCTAAGCTC
ATGAGTCTGTCTCCCATCCAAATGACAGTTTGTCTAAGATCATTAATTTGATTTGCCAATTTTTGATCAATACTAGATTGTGA
ATTCCACAATCTTGTAGAATTTTTTTGCCAATCATTAACAAAGTTTACTGACTGAACAGAAGAGTGCAATGCAACTCCTGCTA
CAGCACCCGTAGCTGTGACTGCAATTAATCCCATAATCACTGCAATTAAAGTAAAAATGAATCTTTTGGATCTATtTAAAACA
CCTTTTAATACTTCAGTCAAAATATGGACGGATGGTGAGGCCTCCCACGGTCGGTCCATGGACACAAGGATCCACACGCCCTC
```

Blast result; best hit: Homo sapiens chromosome 22, GRCh38.p12 Primary Assembly (NC_000022.11); Identities 100%, Gaps 0%:

```
Query  1         GAGAGCACGGGGTTGGGGGTAAGGTTATAGATTAACAGAATCTCAAGGCAGAAGAATTTT  60
                 ||||||||||||||||||||||||||||||||||||||||||||||||||||||||||||
Sbjct  18947033  GAGAGCACGGGGTTGGGGGTAAGGTTATAGATTAACAGAATCTCAAGGCAGAAGAATTTT  18946974
Query  61        TCTTAGCACATAACAAAATGGAGTCTCCTATGTCTACTTCTTTCTACACAGACACAGTAA  120
                 ||||||||||||||||||||||||||||||||||||||||||||||||||||||||||||
Sbjct  18946973  TCTTAGCACATAACAAAATGGAGTCTCCTATGTCTACTTCTTTCTACACAGACACAGTAA  18946914
Query  121       CAATCTGATCTCTCTTGCTTTTCCCCACATTTCCCCCTTTTCTTTTCGACAAAACCGCCA  180
                 ||||||||||||||||||||||||||||||||||||||||||||||||||||||||||||
Sbjct  18946913  CAATCTGATCTCTCTTGCTTTTCCCCACATTTCCCCCTTTTCTTTTCGACAAAACCGCCA  18946854
Query  181       TCGTCATCATGGCCCGTTCTCGATGGTCGCTGTCTCTTCGGAGCTGTTGGGTACACCTGC  240
                 ||||||||||||||||||||||||||||||||||||||||||||||||||||||||||||
Sbjct  18946853  TCGTCATCATGGCCCGTTCTCGATGGTCGCTGTCTCTTCGGAGCTGTTGGGTACACCTGC  18946794
Query  241       AGACTAACAACAGACAAAACAGGCACACAAGGATTAATATGAGATTTATAATCGTAGTAC  300
                 ||||||||||||||||||||||||||||||||||||||||||||||||||||||||||||
Sbjct  18946793  AGACTAACAACAGACAAAACAGGCACACAAGGATTAATATGAGATTTATAATCGTAGTAC  18946734
Query  301       TTCCAATGGTCTTAACCCAAGTGACAGGGTTAAGATTTGCGAGGCCATCAGCAACTCCTG  360
                 ||||||||||||||||||||||||||||||||||||||||||||||||||||||||||||
Sbjct  18946733  TTCCAATGGTCTTAACCCAAGTGACAGGGTTAAGATTTGCGAGGCCATCAGCAACTCCTG  18946674
Query  361       CAATTGCCTCAGTTCCTGGCACCAAATTTAAATGGGCTTTTGATGCTTCGAAAATTTGTT  420
                 ||||||||||||||||||||||||||||||||||||||||||||||||||||||||||||
Sbjct  18946673  CAATTGCCTCAGTTCCTGGCACCAAATTTAAATGGGCTTTTGATGCTTCGAAAATTTGTT  18946614
Query  421       CTTTTAATTTGGAAATGTCTAAAGTGAGATTATCTTCTCTTCCCTGTAGATGGCGTCTAA  480
                 ||||||||||||||||||||||||||||||||||||||||||||||||||||||||||||
Sbjct  18946613  CTTTTAATTTGGAAATGTCTAAAGTGAGATTATCTTCTCTTCCCTGTAGATGGCGTCTAA  18946554
Query  481       CCATGTCCCAGTGATGCTCAGACTCATTATAAATTTGGGGTGTAATACAAAAATCTGACG  540
                 ||||||||||||||||||||||||||||||||||||||||||||||||||||||||||||
Sbjct  18946553  CCATGTCCCAGTGATGCTCAGACTCATTATAAATTTGGGGTGTAATACAAAAATCTGACG  18946494
Query  541       TATTCCAGTCACATTGTAACTGGAAACGATGTTCTAAGCTCATGAGTCTGTCTCCCATCC  600
                 ||||||||||||||||||||||||||||||||||||||||||||||||||||||||||||
Sbjct  18946493  TATTCCAGTCACATTGTAACTGGAAACGATGTTCTAAGCTCATGAGTCTGTCTCCCATCC  18946434
Query  601       AAATGACAGTTTGTCTAAGATCATTAATTTGATTTGCCAATTTTTGATCAATACTAGATT  660
                 ||||||||||||||||||||||||||||||||||||||||||||||||||||||||||||
Sbjct  18946433  AAATGACAGTTTGTCTAAGATCATTAATTTGATTTGCCAATTTTTGATCAATACTAGATT  18946374
Query  661       GTGAATTCCACAATCTTGTAGAAttttttGCCAATCATTAACAAAGTTTACTGACTGAA  720
                 ||||||||||||||||||||||||||||||||||||||||||||||||||||||||||||
Sbjct  18946373  GTGAATTCCACAATCTTGTAGAATTTTTTGCCAATCATTAACAAAGTTTACTGACTGAA  18946314
Query  721       CAGAAGAGTGCAATGCAACTCCTGCTACAGCACCCGTAGCTGTGACTGCAATTAATCCCA  780
                 ||||||||||||||||||||||||||||||||||||||||||||||||||||||||||||
Sbjct  18946313  CAGAAGAGTGCAATGCAACTCCTGCTACAGCACCCGTAGCTGTGACTGCAATTAATCCCA  18946254
Query  781       TAATCACTGCAATTAAAGTAAAAATGAATCTTTTGGATCTATTTAAAACACCTTTTAATA  840
                 ||||||||||||||||||||||||||||||||||||||||||||||||||||||||||||
Sbjct  18946253  TAATCACTGCAATTAAAGTAAAAATGAATCTTTTGGATCTATTTAAAACACCTTTTAATA  18946194
Query  841       CTTCAGTCAAAATATGGACGGATGGTGAGGCCTCCCACGGTCGGTCCATGGACACAAGGA  900
                 ||||||||||||||||||||||||||||||||||||||||||||||||||||||||||||
Sbjct  18946193  CTTCAGTCAAAATATGGACGGATGGTGAGGCCTCCCACGGTCGGTCCATGGACACAAGGA  18946134
Query  901       TCCACACGCCCTC  913
                 |||||||||||||
Sbjct  18946133  TCCACACGCCCTC  18946121
```

Blast result; top hits:

Chromosome	Start	Stop	identities
NC_000022.11	18947033	18946121	913/913
NC_000007.14	4583245	4584157	908/913
NC_000001.11	75382639	75381727	907/913
NC_000005.10	156658525	156659437	906/913
NC_000011.10	101703709	101702797	904/913
NC_000008.11	139460725	139461637	903/913

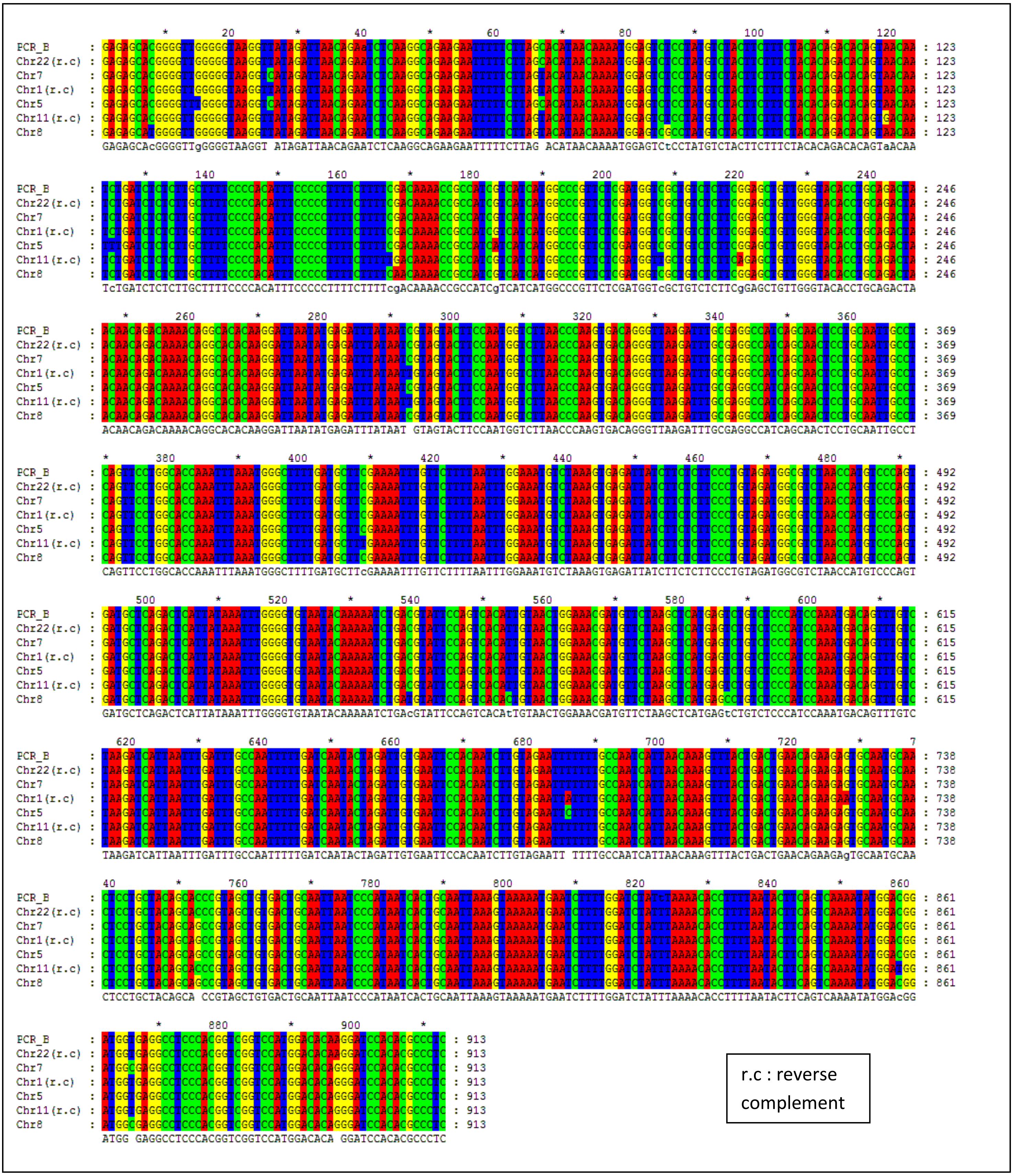

Supplement: Supplementary file 1 [file Data_Sheet_1.pdf]
